# Comparative time-course transcriptome analysis of two contrasting alfalfa (*Medicago sativa* L.) genotypes reveals tolerance mechanisms to salt stress

**DOI:** 10.3389/fpls.2022.1070846

**Published:** 2022-12-08

**Authors:** Dongmei Ma, Jinjun Cai, Qiaoli Ma, Wenjing Wang, Lijuan Zhao, Jiawen Li, Lina Su

**Affiliations:** ^1^ Breeding Base for State Key Laboratory of Land Degradation and Ecological Restoration in Northwest China, Ningxia University, Yinchuan, China; ^2^ Ministry of Education Key Laboratory for Restoration and Reconstruction of Degraded Ecosystems in Northwest China, Ningxia University, Yinchuan, China; ^3^ Key Laboratory of Modern Molecular Breeding for Dominant and Special Crops in Ningxia, Ningxia University, Yinchuan, China; ^4^ Institute of Agricultural Resources and Environment, Ningxia Academy of Agriculture and Forestry Sciences, Yinchuan, China; ^5^ Agricultural College, Ningxia University, Yinchuan, China

**Keywords:** alfalfa, transcriptome, salt tolerance, post-translational modification, signal sensing and transduction

## Abstract

Salt stress is a major abiotic stress affecting plant growth and crop yield. For the successful cultivation of alfalfa (*Medicago sativa* L.), a key legume forage, in saline-affected areas, it’s essential to explore genetic modifications to improve salt-tolerance.Transcriptome assay of two comparative alfalfa genotypes, Adina and Zhaodong, following a 4 h and 8 h’s 300 mM NaCl treatment was conducted in this study in order to investigate the molecular mechanism in alfalfa under salt stress conditions. Results showed that we obtained 875,023,571 transcripts and 662,765,594 unigenes were abtained from the sequenced libraries, and 520,091 assembled unigenes were annotated in at least one database. Among them, we identified 1,636 differentially expression genes (DEGs) in Adina, of which 1,426 were up-regulated and 210 down-regulated, and 1,295 DEGs in Zhaodong, of which 565 were up-regulated and 730 down-regulated. GO annotations and KEGG pathway enrichments of the DEGs based on RNA-seq data indicated that DEGs were involved in (1) ion and membrane homeostasis, including ABC transporter, *CLC*, *NCX*, and *NHX*; (2) Ca^2+^ sensing and transduction, including BK channel, EF-hand domain, and calmodulin binding protein; (3) phytohormone signaling and regulation, including *TPR, FBP, LRR*, and *PP2C*; (4) transcription factors, including zinc finger proteins, *YABBY*, and SBP-box; (5) antioxidation process, including *GST*, PYROX, and *ALDH*; (6) post-translational modification, including *UCH*, ubiquitin family, *GT, MT* and *SOT*. The functional roles of DEGs could explain the variations in salt tolerance performance observed between the two alfalfa genotypes Adina and Zhaodong. Our study widens the understanding of the sophisticated molecular response and tolerance mechanism to salt stress, providing novel insights on candidate genes and pathways for genetic modification involved in salt stress adaptation in alfalfa.

## Introduction

Salt stress is a key abiotic stress threatening germination, growth, development, and seed set formation of plants (Munns and Tester, 2008). It’s crucial to improve plants’ endurance to salinity in order to support plant growth and crop yield. Plants undergo a number of morphological, cellular, physiological, biochemical, and molecular changes when coping with salt stress, and they have evolved sophisticated tolerance arrangements to defend themselves from salt stress conditions (Parihar et al., 2015; Haak et al., 2017). These adjustments are regulated by multiple genes and molecular mechanisms. Therefore, it is imperative to identify and understand the genes and molecular mechanisms involved in salt stress responses in order to improve salt resistance capacity in plants, which would subsequently make great contributions for the genetic modification of crops and food production in saline affected areas.

During the past decades, key molecular mechanisms mediated plant salt response and tolerance have been identified. We know that the hyperosmotic component and the ionic Na^+^ component caused by salinity are perceived by the salt stress sensing and signaling components (e.g. reactive oxygen species (ROS), abscisic acid (ABA), Ca^2+^ response, and kinases (calcium-dependent protein kinases (CDPKs), and calcineurin B-like proteins with CBL-interacting protein kinases (CIPKs), which would transduce the signal of hyperosmotic pressure (Deinlein et al., 2014; Dinneny, 2015; You and Chan, 2015). The salt stress sensory and signaling mechanisms to achieve tolerance can be integrated by the linkage of transcription factors in plants. These transcription factors include calmodulin-binding transcription activators (CAMTAs), GT element-binding like proteins (GTLs), basic leucine zipper (bZIP), WRKY, MYB, APETALA2/ETHYLENE RESPONSE FACTOR (AP2/ERF), NAC, and basic helix–loop–helix (bHLH) families (Deinlein et al., 2014; Rasul et al., 2017; Li et al., 2018; Sun et al., 2018; Wang et al., 2018; Büyük et al., 2019), which can regulate the transcript expression patterns of genes involved in salt response and tolerance in plants. In addition, salt overly sensitive (SOS) in maintaining low Na^+^ in the cytoplasm, HKT in Na^+^ partitioning, and some other additional regulators have been reported to be intimately associated with salt tolerance in plants (Deinlein et al., 2014). Yet, salt tolerance mechanisms remained elusive due to the sophisticated gene regulation network and the genetic variability among plant species.

In recent years, next-generation sequencing (NGS) technologies have been broadly adopted for their high precision and throughput to explore the molecular mechanism of salt response and tolerance in plants. It is worth clarifying that transcriptome studies have used RNA sequencing approaches not only in model plants and important crops like rice (*Oryza sativa*) (Shankar et al., 2016), maize (*Zea mays*) (Du et al., 2017), wild cotton species (*Gossypium klotzschianum*) (Wei et al., 2017), and *Brachypodium distachyon* (Priest et al., 2014), but also in plant species for which no reference genome is available, for instance ryegrass (*Lolium perenne*) (Hu et al., 2016), *Carex rigescens* (Li et al., 2017; Zhang et al., 2020a), and radish (*Raphanus sativus* L.) (Sun et al., 2016). A number of transcripts that play critical roles in salt stress response and tolerance regulation have been identified, largely contributing to the understanding of plant salt tolerance mechanisms. The usage of the RNA sequencing approaches makes the dynamic detection of transcripts in plants under salt stress accessible. Multi-transcriptomes at different time points can be used as comparisons to identify more genes and mechanisms intimately linked to plant salt stress tolerance, as studies have done in *Carex rigescens* (Zhang et al., 2020a), potato (*Solanum tuberosum* L.) (Li et al., 2020), sorghum (*Sorghum bicolor* L.) (Cui et al., 2018), and soybean (*Glycine max* L.) (Liu et al., 2019a). Yet the regulatory network involved in dynamic salt response is still not fully understood. Transcriptome analyses of different plant species should be considered the preferred way to reveal salt response and tolerance systems in plants.

Alfalfa (*Medicago sativa* L.), an important perennial forage legume, is widely cultivated across the world and it’s valued for its high protein content, nutritional value, stress resistance capabilities, and biomass production (Luo et al., 2014). The mainly areas where alfalfa is cultivated in the northwestern, northeastern, and northern coastal regions of China, are unfortunately affected by increased soil salinization, which hinders the growth and production of alfalfa plants (Ashrafi et al., 2014). As such it is vital to understand salt tolerance mechanisms in alfalfa. Research on the molecular mechanisms involved in salt stress adaptation and tolerance in alfalfa has been progressing: (1) large-scale dissection of transcripts, proteins, metabolites, and genetic loci have been identified *via* transcriptome (Long et al., 2015), proteome (Long et al., 2016; Long et al., 2018) and genome-wide association analyses (Yu et al., 2016); (2) a number of genes and regulators involved in salt tolerance in alfalfa, such as *MsGRP* (Long et al., 2013), *MsZEP* (Zhang et al., 2016), miR393 (Long et al., 2017) and miR156 (Arshad et al., 2017), have been identified by molecular function assays; (3) it has been observed that the overexpression of salt tolerance genes such as *AgcodA* (Li et al., 2014) and *AtNDPK2* (Wang et al., 2014), and the co-overexpression of *ZxNHX* and *ZxVP1-1* (Kang et al., 2016) improve salt stress adaptation in alfalfa. Nevertheless, research on the integrated signaling pathways and regulatory networks involved in the response to salt stress in alfalfa is still limited, and understanding of the mechanisms and the genetic modifications of alfalfa necessary to salt stress tolerance is still a challenge.

In this study, we hypothesize that the salt response and tolerance mechanisms can be revealed by time-course transcriptional comparison between two *Medicago sativa* genotypes (Adina and Zhaodong), which were previously screened as contrast salt responsive genotypes by physiological tests. We perform gene expression analysis and identify differentially expressed genes in salt stress conditions, subsequently we carry out GO annotation and KEGG pathway enrichment analysis. Our aim is to achieve a wide view of the transcriptional expression of genes in salt stress response and to reveal salt tolerance genes and mechanisms in alfalfa. In addition, we explore the genetic variation at transcriptional level of two salt tolerance comparable alfalfa genotypes.

## Materials and methods

### Plant materials and treatment conditions

Two alfalfa (*Medicago sativa*) cultivars (cvs. Adina, Zhaodong) were used in this study. Our growth and physiological assessments noted that Adina is salt-tolerant, while Zhaodong is salt-sensitive (**Figure S1**). Alfalfa seeds were immersed in 75% ethanol for 30 seconds and then sterilized by 0.1% HgCl_2_ solution for 8 minutes. After rinsing with double distilled water 5 times, the seeds were let germinate on filter paper on sterilized petri dishes. Alfalfa seed germination was achieved in a growth chamber under conditions of 16-h-light (1200 μmol m^−2^ s^−1^)/8-h-dark, 25°C temperature, and 85% humidity. Five alfalfa seedlings in similar growth conditions were transferred in a tube (13 cm×5 cm) containing half-strength Hoagland’s nutrient solution for hydroponic culture. The nutrient solution was replaced every two days to keep it fresh.

Thirty-day-old seedlings were subjected to salt treatment by transferring them to 300 mM NaCl nutrient solution. The root was chosen for transcriptome analysis as it’s the first organ to experience salt stress (Postnikova et al., 2013). The roots of Adina and Zhaodong were harvested at 4 h and 8 h after salt treatment and rapidly rinsed with double distilled water. The roots of Adina and Zhaodong without NaCl addition were sampled as control. Three individual plants were collected as one sample, and three biological replicates were conducted in this study. The harvested samples were flash-frozen with liquid nitrogen and preserved at -80°C. N0, N4, and N8, were used to indicate the Adina root samples respectively at 0, 4 h, and 8 h after salt treatments, and M0, M4, and M8 for Zhaodong samples at 0, 4 h, and 8 h after salt treatments.

### Total RNA isolation and sequencing

Total RNA in the alfalfa root samples was isolated using Trizol Reagent (Invitrogen, Beijing, China) following the manufacturer’s instruction. The RNA quality was examined on 1% RNase free agarose gel, the purity was confirmed by a Nanodrop-2000 spectrophotometer (Thermo Scientific, DE, USA), the integrity was determined by an Agilent Bioanalyzer 2100 System (Agilent Technologies, CA, USA), and the precise concentration was determined by a Qubit 2.0 Fluorimeter (Life Technologies, CA, USA). The sequencing library was constructed using a NEBNext^®^ Ultra^™^ RNA Library Prep Kit (NEB, USA) according to the manufacturer’s recommendations and index codes. After verifying the quality of the cDNA library obtained, the samples were sequenced with the Illumina HiSeq/MiSeq sequencer.

### *De novo* assembly and functional annotation

Raw reads, which have been submitted to the SRA public database (accession: PRJNA821982), were cleaned removing adapter sequences, low-quality sequences, reads with ambiguous bases ‘N’, and reads with more than 10% bases with Q< 20. The high-quality clean data was used for the *de novo* assembly by Trinity assembler (http://trinityrnaseq.sourceforge.net/) (Grabherr et al., 2011). The functional annotations of the assembled unigenes were compared to the databases: non-redundant protein sequences (Nr); non-redundant nucleotide sequences (Nt); protein family (Pfam); eukaryotic Ortholog Groups/Clusters of Orthologous Groups of proteins (KOG/COG) and a manually annotated and reviewed protein sequence database (Swiss-Prot). The KEGG (Kyoto Encyclopedia of Genes and Genomes) pathways analysis annotation was obtained with the KEGG Automatic Annotation Server (KAAS). The Gene Ontology (GO) annotation of unigenes was accessed by Blast2GO program (v2.5) according to the Nr and Pfam annotation results.

### Differential unigene expression analysis

The clean reads were mapped back to the assembled sequences with Bowtie2 in RSEM software (Li and Dewey, 2011). The read counts were converted to FPKM (Fragments Per Kilobase Million) to represent the gene expression level. The differential expression of the unigenes, which was generated with the Benjamini and Hochberg’s approach, was determined by DESeq R package with adjusted *P* value< 0.05 and the criteria of |log2FC| > 1. The GO enrichment analysis was conducted with the GO-seq and topGO R packages. The KEGG analysis was carried out with the KOBAS software.

### Quantitative real-time PCR validation

Seven genes were randomly selected for quantitative RT-PCR analysis in order to validate the RNA-seq data. RNA samples of alfalfa roots were reverse transcribed into cDNA using a PrimeScript™ RT reagent kit with gDNA Eraser (Takara, China). The amplification of the candidate genes was conducted on an ABI qPCR System (Applied biosystems, United States) with SYBR Premix Ex Taq (TliRNaseH Plus) (Takara, China). The PCR cycling reaction procedure was 95°C for 30 s, followed by 42 cycles of 95°C for 30 s, and 55°C for 15 s. After each PCR run a dissociation curve was generated to confirm the specificity of the product. The primers for qRT-PCR validation were designed using Beacon Designer software (version 7.8) and are listed in **Table S1**. The *β-ACTIN* gene of *M. sativa* was used as the internal control for gene expression normalization. The expression level of the candidate genes was calculated with the 2^−ΔΔCt^ method.

## Results

### Transcriptome sequencing and assembly

We sequenced eighteen cDNA libraries of two alfalfa cultivars. We acquired raw reads for each library by PCR and subsequent Illumina sequencing, we then obtained the clean reads removing ambiguous nucleotides, adapter sequences, and low-quality sequences. The raw reads, clean reads, and the quality assessments of the eighteen libraries are shown in **Table S2**. From the 18 sequenced libraries we obtained in total 875,023,571 transcripts with a mean length of 623 bp, an N50 of 946 bp, and an N90 of 256 bp, and 662,765,594 unigenes with a mean length of 1,014 bp, an N50 of 1,353 bp, and an N90 of 491 bp (**Table 1**). The transcripts and unigenes were grouped by length as shown in **Table 1**.

**Table 1:** A Summary of assembled transcripts and unigenes for all samples.

	Transcripts	Unigenes
**Min Length**	201	201
**Mean Length**	623	1,014
**Median Length**	348	718
**Max Length**	20,230	20,230
**Number of N50**	946	1,353
**Number of N90**	256	491
**Total Nucleotides**	875,023,571	662,765,594
**Number of length (bp) <301**	45,822	581,806
**Number of length (bp): 301-500**	144,183	341,594
**Number of length (bp): 501-1000**	241,797	258,808
**Number of length (bp): 1001-2000**	152,105	152,272
**Number of length (bp): >2000**	69,556	69,556

N50, the size of the transcripts or the unigenes above which the assembly contains at least 50% of the total length of all the contigs; N90, the size of the transcripts or the unigenes above which the assembly contains at least 50% of the total length of all the contigs.

### Unigenes annotations and pathway assignments

In order to explore their integrated function, we blasted the unigenes against seven databases (NR, NT, KO, SwissProt, PFAM, GO, KOG) for similarity searches. The numbers of the annotated unigenes and the percentage of the total are listed in **Table 2**. 520,091 assembled unigenes, 79.58% of the total, were annotated in at least one database.

**Table 2:** Blast analysis of assembled unigenes.

	Number of Genes	Percentage (%)
**Annotated in NR**	356,535	54.56
**Annotated in NT**	278,059	42.55
**Annotated in KO**	159,597	24.42
**Annotated in SwissProt**	311,429	47.65
**Annotated in PFAM**	347,366	53.15
**Annotated in GO**	356,281	54.52
**Annotated in KOG**	164,855	25.22
**Annotated in all Databases**	48,407	7.4
**Annotated in at least one Database**	520,091	79.58
**Total Unigenes Annotated**	653,466	100

Among them, 356,281 (54.52%) assembled unigenes showed identity in the GO database. The matched unigenes could be sorted into three categories (biological processes, cellular components, and molecular functions), and further classified in 56 subcategories (**Figure S2A**). The three main subcategories within the category of biological processes were cellular process, metabolic process, and single-organism process. Cell and cell part were the two dominant subcategories in cellular components, while binding and catalytic activity were the major subcategories within molecular functions.

When analyzed in the KEGG database, 159,597 unigenes were found to belong to five classifications and to be involved in 19 pathways: 6.6% (10,576) in cellular processes, 3.5% (5,524) in environmental information processing, 26.5% (42,290) in genetic information processing, 45.1% (71,943) in metabolism, and 2.8% (4,462) in cellular processes, while translation (18,770), carbohydrate metabolism (14,340), folding, sorting and degradation (13,063), overview (11,543), and transport and catabolism (10,576) were the dominant pathways (**Figure S2B**).

### Expression and cluster analysis of the genes

**Figure 1:**
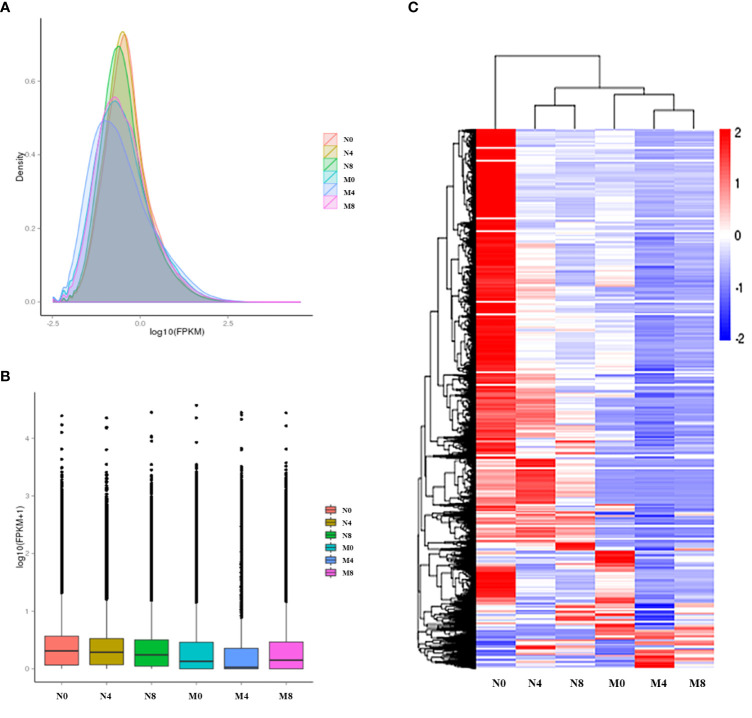
Expression analysis of the genes. N0, N4, and N8, represent the alfalfa root samples of Zhaodong respectively after 0 h, 4 h, and 8 h of salt treatment. M0, M4, and M8, represent the alfalfa root samples of Adina respectively after 0 h, 4 h, and 8 h of salt treatment. **(A)** FPKM density distribution. **(B)** FPKM distribution. **(C)** Hierarchical cluster of genes in the six alfalfa samples. The colors represent log2(FPKM value) in each grid, the color key is on the right.

In order to analyze the expression level of genes, we converted the read counts of alfalfa transcriptome to FPKM, as its density distribution can reflect the integrated gene expression pattern of each sequenced sample. Some of the gene expression levels in the salt-tolerant samples (N0, N4, N8) were higher than in the salt-sensitive samples (M0, M4, M8) (**Figure 1**). To estimate the gene expression at different times in Adina and Zhaodong, we hierarchically clustered the genes in the six samples with their log2(ratios) values. Results showed that most of the genes were differentially expressed after salt treatment in Adina and Zhaodong and at 4 h and 8 h after salt treatment (**Figure 1C**).

### Differential expression analysis of the genes

**Figure 2:**
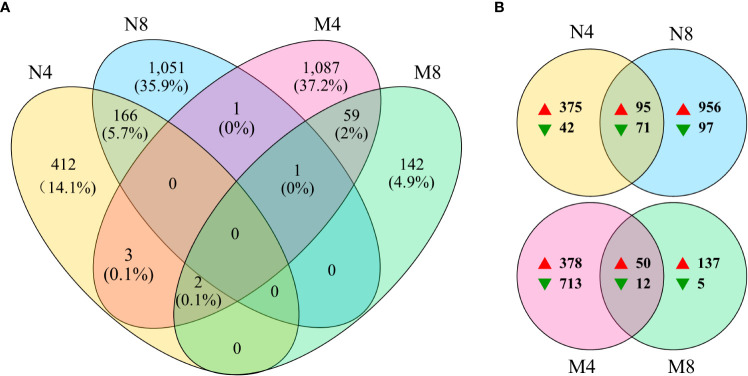
Venn diagram of the four comparisons. **(A)** The overlapped numbers of genes in the four groups. **(B)** The number of up-regulated (on the right of the red arrows) and down-regulated (on the right of the green arrows) genes in the four groups. N4 and N8 represent the comparison groups of the genes identified at 4 h and 8 h after treatment to 0 h in Adina. M4 and M8 represent the comparison groups of the genes identified at 4 h and 8 h after treatment to 0 h in Zhaodong.

The numbers, changes, overlaps, and the ratios to total, of the DEGs identified in the four comparison groups (N4, N8, M4, M8) were exhibited in the Venn gram (**Figure 2**). After salt treatment we found 583 DEGs in N4 and 1,219 in N8, with an overlap of 166 DEGs. We identified 1,153 and 204 DEGs, with an overlap of 62, respectively in M4 and M8. In addition, we found a total of 1,636 DEGs, 1,426 up-regulated and 210 down-regulated, in the salt-tolerant cultivar Adina, and a total of 1,295 DEGs, 565 up-regulated and 730 down-regulated, in the salt-tolerant cultivar Zhaodong. The fact that more DEGs are upregulated in Adina compared to Zhaodong could indicate the induction of the transcriptional regulation involved in molecular salt-tolerance in Adina.

### GO annotations of the DEGs

**Figure 3:**
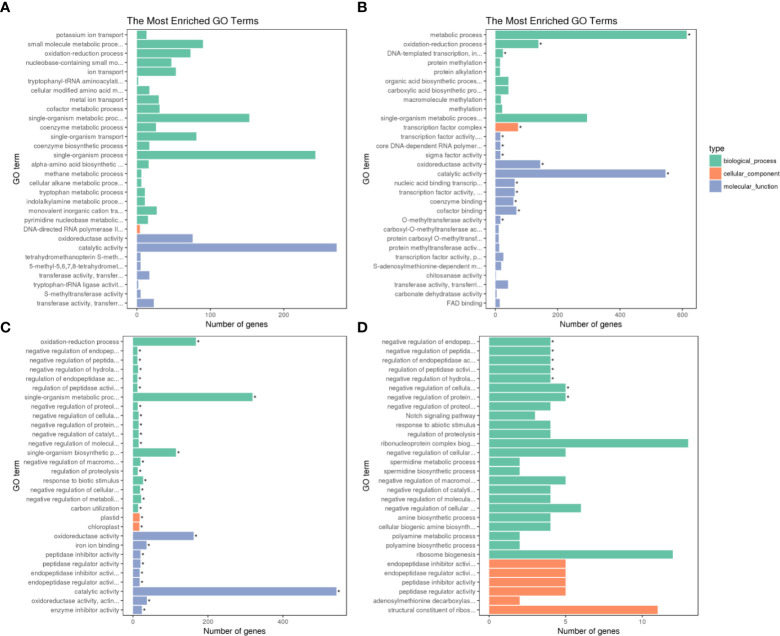
GO categorization of the DEGs. DEGs were separated into metabolic categories based on biological processes, cellular components and molecular functions. GO categorization of the DEGs in N4 **(A)**, N8 **(B)**, M4 **(C)**, and M8 **(D)**. N4 and N8 represent the comparison groups of the genes identified at 4 h and 8 h after treatment in Adina. M4 and M8 represent the comparison groups of the genes identified at 4 h and 8 h after treatment to 0 h in Zhaodong. *Asterisk means DEGs were significantly enriched in this GO term.

The GO annotation classification of the DEGs in Adina and Zhaodong were displayed in **Figure 3**. The DEGs were categorized into three metabolic categories: biological processes, cellular components, and molecular functions. In biological processes, the subcategory of oxidation-reduction was highly enriched in both Adina and Zhaodong, while the metabolic process was significantly enriched only in Adina. In terms of cellular components, the transcription factor complex was enriched only in Adina, while plastid and chloroplast were only significantly enriched in Zhaodong. In the category of molecular functions, the most abundant subcategories in both Adina and Zhaodong were catalytic activity and oxidoreductase activity in. Annotations such as transcription factor activity, cofactor binding, coenzyme binding, nucleic acid binding transcription, were significantly enriched in Adina, whereas processes such as enzyme inhibitor activity and peptidase inhibitor activity were enriched in Zhaodong.

### KEGG pathway enrichment of the DEGs

**Figure 4:**
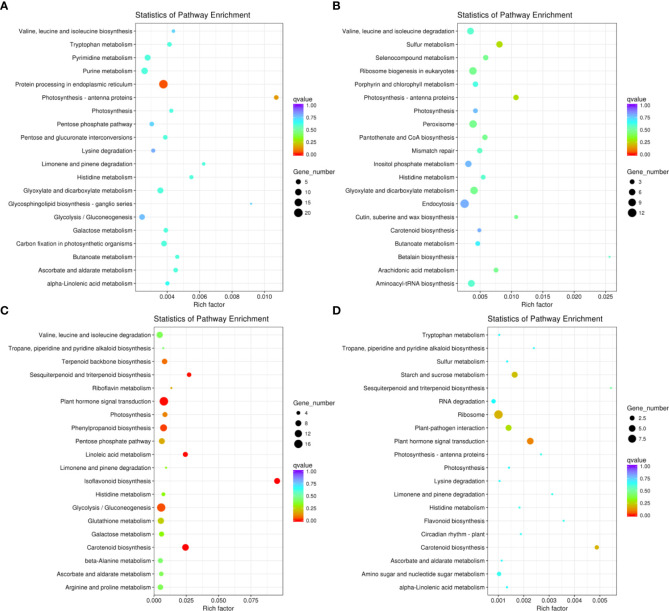
KEGG functional classification of the DEGs. The sizes of the spots represent the number of genes, and the colors represent the q-value. KEGG functional classification of the DEGs in N4 **(A)**, N8 **(B)**, M4 **(C)**, and M8 **(D)**. N4 and N8 represent the comparison groups of the genes identified at 4 h and 8 h after treatment to 0 h in Adina. M4 and M8 represent the comparison groups of the genes identified at 4 h and 8 h after treatments to 0 h in Zhaodong.

We conducted KEGG pathway analysis of the DEGs in Adina and Zhaodong (**Figure 4**). We found that DEGs in Adina were mainly enriched in photosynthesis-antenna proteins, protein processing in endoplasmic reticulum, glyoxylate and dicarboxylate metabolism, and pentose and glucuronate interconversions pathways. In Zhaodong DEGs were enriched in plant hormone signal transduction, photosynthesis, starch and sucrose metabolism, pentose phosphate pathway, glycolysis/gluconeogenesis, and glutathione metabolism pathways. The KEGG enrichments in Adina and Zhaodong subjected to salt stress indicate that the biological processes of photosynthesis, energy generation and usage, signal sensing and transduction, and protein synthesis are modified in salt stress conditions.

### RNA-seq data validation with qRT-PCR

We randomly chose **s**even DEGs to validate the RNA-seq data by qRT-PCR assay. The correlation analysis between RNA-seq data and qRT-PCR are presented in **Table 3**. The correlation coefficients are greater than 0.6, showing a high correlation between the data of RNA-seq and qRT-PCR. The results of the correlation analysis indicate that RNA-seq data is reliable and can be used for further functional analysis.

**Table 3:** Correlation coefficient between RNA-seq data and qRT-PCR.

Gene ID	Correlation coefficient
**Cluster-171808.189526**	0.8
**Cluster-171808.190107**	0.731
**Cluster-171808.201554**	0.819
**Cluster-171808.198991**	0.886
**Cluster-171808.81350**	0.933
**Cluster-171808.262653**	0.616
**Cluster-171808.116680**	0.782

## Discussions

**Figure 5:**
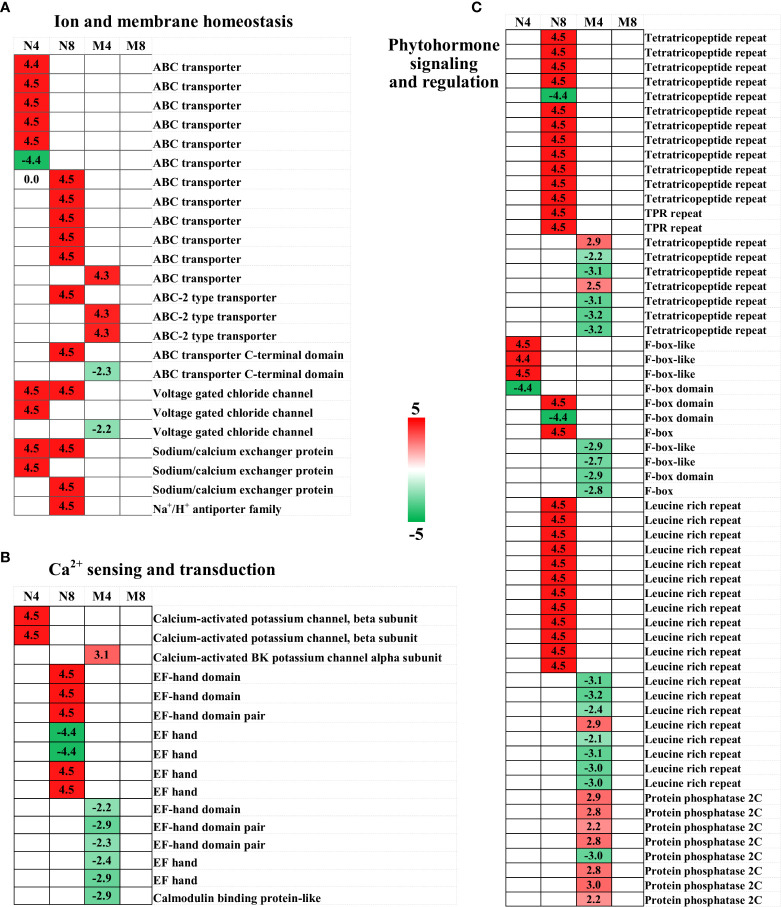
Heat map of candidate DEGs involved in ion and membrane homeostasis **(A)**, antioxidation process **(B)**, and phytohormone signaling and regulation **(C)**. The four columns, from left to right, represent N4, N8, M4, and M8. Color key is shown on the right. Red represents up-regulation, green down-regulation, and white no significant change.

**Figure 6:**
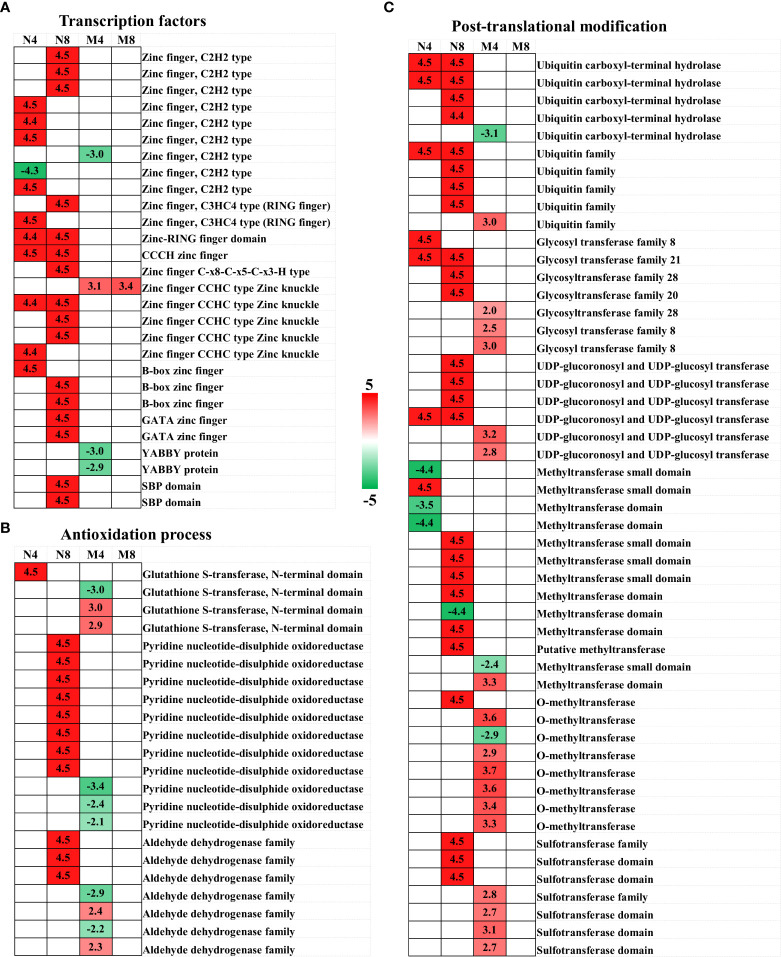
**Heat map of candidate DEGs** involved in transcription factors **(A)**, Ca^2+^ sensing and transduction **(B)**, and post-translational modification **(C)**. Samples values used are log_2_(fold change). The four columns, from left to right, represent N4, N8, M4, and M8. Color key is shown on the right. Red represents up-regulation, green down-regulation, and white no significant change.

In this study we conducted a dynamic transcriptome survey of two alfalfa genotypes to investigate the transcriptional changes happening in alfalfa in response to salt stress conditions. From the sequenced samples we obtained more than 87 million transcripts and 66 unigenes. In addition, we performed differential gene expression analysis, GO annotation and KEGG enrichment analysis of the unigenes and DEGs, generating clues about the salt response mechanism of alfalfa. We revealed a dynamic and wide perspective of salt adaptation and tolerance changes at the transcriptional level through the comparison of DEGs expression in two alfalfa genotypes with significant salt tolerance genetic variations. We will discuss specific DEGs involved in ion and membrane homeostasis, Ca^2+^ sensing and transduction, phytohormone signaling and regulation, transcription factors, antioxidation process, post-translational modification (**Figures 5**, **6**, **Table S3**) contributing to salt stress adaptation in alfalfa in the next sections of this paper.

### DEGs involved in ion and membrane homeostasis

In plants the negative electrical potential caused by salt stress can occur through Na^+^ influx in ion channels or other membrane transport proteins, which can enhance the passive diffusion of Na^+^ across the plasma membrane. As such, sodium and chloride channels and transporters are essential for salt stress defense processes (Amtmann and Beilby, 2010). In our study, we identified as DEGs after salt treatment 12 ATP-binding cassette (ABC) transporter genes, which are involved in the ATP-powered export or import of various substrates across biological membranes and act as important membrane transporters in plants (Kang et al., 2010; Moon and Jung, 2014). 11 of the 12 ABC genes were up-regulated, consistently with a prior transcriptome study (Wu et al., 2017). The enhanced expression of the ABC transporters could contribute to salt resistance reducing the passage and transport of sodium (Kang et al., 2010; Kim et al., 2010; Wu et al., 2017). We also identified as DEGs three ABC-2 type transporters and two ABC transporter C-terminal domain, suggesting ABC-transporters could have a role in enabling alfalfa to cope with salt stress through ion signaling and transportation. In addition, we noticed two voltage gated chloride channel (*CLC*) genes, three sodium/calcium exchanger (*NCX*) genes, and a Na^+^/H^+^ antiporter family (*NHX*) gene were upregulated in Adina, while one *CLC* was downregulated in Zhaodong. *CLC*, *NCX*, and *NHX* are known to play important roles in intracellular Na^+^ and Cl^-^ transportation and homeostasis under salt stress conditions and are considered important gene targets for salt tolerance improvement in plants (Wu et al., 2004; Wang et al., 2012; Wei et al., 2013; Liu et al., 2020). The up-regulation changes observed in Adina indicate the role of the genes in salt tolerance in alfalfa.

In salt stress conditions plants uptake macronutrients such as K^+^, ${\text{NO}}_{3}^{-}$ and Ca^2+^ to mitigate Na^+^ and Cl^-^ assimilation (Amtmann and Beilby, 2010). It has been proposed that *CLC* could regulate the transport, interaction and homeostasis of ${\text{NO}}_{3}^{-}$ in cotton (*Gossypium hirsutum* L.), in addition to its Cl^-^ transportation role (Liu et al., 2020). We therefore suggest that the ion and membrane homeostasis in alfalfa in salt stress conditions could be maintained by the identified DEGs, especially in Adina, which shows upregulated sodium and chloride channels and transporters genes.

### DEGs involved in Ca^2+^ sensing and transduction

Ca^2+^ is a ubiquitous second messenger that plays a critical role in plant responses to salinity (Zeng et al., 2017). We identified three upregulated calcium-activated potassium channel genes (BK channel), two in Adina and one Zhaodong. It has been proposed that BK channels are activated by Ca^2+^ waves and can be coupled by the Ca^2+^ spark (Jonathan Ledoux et al., 2006), therefore the up-regulation of BK channels in Adina could help to hyperpolarizing of the cell membrane for Ca^2+^ sensing and control (Jonathan Ledoux et al., 2006; Hadi and Karimi, 2012). In our study we identified twelve EF-hand domain genes as DEGs, with five upregulated in Adina and none upregulated in Zhaodong. The structural properties of the EF-hand domain allow a rapid response to cytosolic Ca^2+^ concentration and on/off Ca^2+^ binding, and the majority of the key Ca^2+^ sensors and transducers contain the EF-hand domain (Zeng et al., 2017). In rice overexpression of *OsCCD1*, a small calcium-binding protein with one EF-hand motif, can significantly enhance tolerance to salt stress (Jing et al., 2016), while a different study showed how Ca^2+^ binding to the EF-hand domain of *ZmNSA1* can improve salt-tolerance in maize by increasing SOS1 Na^+^/H^+^ antiporter-mediated root Na^+^ efflux (Cao et al., 2020). Consistently with these findings, we also found that the SOS1 Na^+^/H^+^ antiporter family (*NHX*) was up-regulated and modified at the EF-hand domain in Adina, supporting the evidence that Ca^2+^ sensing and signaling play an important role in salt-tolerance in Adina (Cao et al., 2020). At the same time the downregulation of the calmodulin binding protein observed in Zhaodong, together with the down-regulation of the five genes encoding EF-hand domain in Zhaodong, suggests that the Ca^2+^ sensing and transduction processes were not enhanced as in Adina.

### DEGs involved in phytohormone signaling and regulation

A number of phytohormonal signals work together in cellular physiology to enable the plant to adapt to the salinity of the environment (Liu et al., 2012). In our study, we identified 21 tetratricopeptide repeats (*TPR*) genes known to be involved in plant salt stress response *via* hormone signaling, 13 genes were up-regulated in Adina and 2 were up-regulated in Zhaodong (Sharma and Pandey, 2016). *TPR*s expression have been proved to be induced under salt stress in rice (Wang et al., 2016b) and Hulless barley (*Hordeum vulgare* L.) (Lai et al., 2020). TPRs regulate salt stress signals *via* gibberellin and cytokinin cross-talk and ABA biosynthesis and transport. In accord with these results, in our study we observed that the induction of *TPR* in Adina in response to salt stress could upregulate plant hormones, such as ABA, GA, cytokinin, and ethylene signaling pathways (Sharma and Pandey, 2016; Wang et al., 2016b; Lai et al., 2020). In addition, we identified ten F-box protein (*FBP*) genes as DEGs after salt stress treatment, with five upregulated in Adina, and none upregulated in Zhaodong. *FBPs*, for example *ARKP1* in *Arabidopsis* (Li et al., 2016), or *TaFBA1* in wheat (*Triticum aestivum*) (Kong et al., 2016), have been documented to be positive regulators for ABA signaling, and plants overexpressing *FBP* present a higher stress-tolerance and a higher antioxidant ability (Abd-Hamid et al., 2020). All of the twelve leucine rich repeat (*LRR*) genes identified in Adina were up-regulated after salt stress treatment, whereas seven of the eight identified in Zhaodong were downregulated. Leucine-rich repeats receptor-like kinases (LRR-RLKs) are known to play important roles in plant defense to salt stress through the regulation of salt stress-related and ABA-depending signaling pathways (Wang et al., 2016a). The increased expression of *FBP* and *LRR* could indicate that the ABA-depending signaling pathway is enhanced to improve salt stress sensing and signaling in Adina. We identified eight protein phosphatase 2C (PP2C) genes, which are regulators of ABA early signal transduction, as DEGs with seven increased and one decreased in Zhaodong. Over-expression of *AtPP2CG1* can improve salt tolerance, enabling *PP2C* to move from a repressor-associated suppression status to an activator-mediated transcription status in response to salt stress (Liu et al., 2012; Nguyen et al., 2019), suggesting PP2C has a regulation role in salt stress adaptation in Zhaodong.

### DEGs encoding transcription factors

Transcription factors (TFs) play important roles in plant adaptation to salt stress regulating downstream genes responsible for salt-tolerance (Sun et al., 2010). In this study, we found that 25 zinc finger protein genes, part of one of the largest transcription factor families in plants, were differentially expressed after salt stress treatments in two alfalfa cultivars. Previous studies have reported these genes (for instance ZFP179 encoding C2H2-type zinc finger protein in rice (Sun et al., 2010), *BrRZFP1* encoding C3HC4-type in *Brassica rapa* (Jung et al., 2013),*GhZFP1* encoding CCCH-type in *Gossypium hirsutum* (Guo et al., 2009), *OsZFP6* encoding CCHC-type in rice (Guan et al., 2014)) play vital roles in the resistance to salt stress by regulating salt-tolerance genes, suggesting the 19 gene encoding these four types of zinc finger protein in alfalfa, with 17 of them up-regulated and two down-regulated, have an important role in salt adaption role. In addition, we observed three genes encoding the B-box zinc finger proteins, and two genes encoding GATA zinc finger proteins were upregulated in Adina. B-box zinc finger proteins and GATA zinc finger proteins have been recently discovered to be tightly associated with ABA signaling and ROS scavenging in the defense from salt stress (Gupta et al., 2017; Liu et al., 2019b), indicating the role these five genes play in Adina. We detected two YABBY transcription factors, part of the subfamily of zinc finger protein, differentially expressed in Zhaodong. *YABBY*s were previously identified as the DEGs in cotton (*Gossypium klotzschianum*) in salt stress condition in a study carried out on the transcriptomic data (Wei et al., 2017). *YABBY*s could be negative regulatory factors in salt stress response in Zhaodong, as *AcYABBY4* does in *Arabidopsis* and *GmYABBY10* in *Glycine max* (Zhao et al., 2017; Li et al., 2019). We identified two up-regulated Squamosa promoter binding protein (SBP)-box genes in Adina, which encode transcription factors exclusive to plants. In previous research it was demonstrated how *Arabidopsis* plants over-expressing an SBP-box transcription factor (*VpSBP16*) from a Chinese wild vitis species had improved salt-tolerance because of the better regulation of SOS and ROS signaling (Hou et al., 2018), indicating *SBPs* may have a role in salt stress defense in Adina.

### DEGs involved in the antioxidation process

Exposure of plants to salt stress leads to oxidative stress and damage (Munns and Tester, 2008). Four glutathione S-transferases (*GSTs*), ubiquitous enzymes playing an important role in cellular detoxification under abiotic stress, were identified as DEGs in our study. Overexpressing *GST*s, as *OsGSTU4* (Sharma et al., 2014) and *LbGST1* (Diao et al., 2011), can significantly reduce the production of the reactive oxygen species and oxidative damage. Three of the four GSTs found in our study were upregulated, indicating their roles in salt stress defense *via* enhancing the antioxidation process in alfalfa. In addition, we found eight pyridine nucleotide-disulphide oxidoreductase genes (*PYROX*), which encode the conserved domain of glutathione reductase (GR), a major antioxidase in plants (Trivedi et al., 2013), were up-regulated in Adina, and three down-regulated in Zhaodong. The resulting up-regulation of the pyridine nucleotide-disulphide oxidoreductase gene could indicate a higher expression of the *GR* transcripts, resulting in more GR activity and therefore oxidation resistance in Adina (Trivedi et al., 2013; Mudalkar et al., 2017). The accumulation of aldehydes under salt stress is another factor that leads to the oxidative stress, and the major detoxification pathways of aldehyde is the oxidation of their carbonyl groups into carboxylic acids by aldehyde dehydrogenase (ALDH) (Xu et al., 2013). We identified seven *ALDH* genes as DEGs after salt stress treatment, three in Adina and four in Zhaodong. The 3 *ALDH* genes in Adina were up-regulated, suggesting the detoxification process in Adina by ALDH may be enhanced in these conditions (Gautam et al., 2020).

### DEGs involved in post-translational modifications

Post-translational modifications play critical roles for plants’ response to salt stress (Zhou et al., 2012). We found four ubiquitin carboxyl-terminal hydrolases (*UCH*), which are involved in the plant ubiquitin C-terminal hydrolases, were increased in Adina, and one decreased in Zhaodong. Genes with UCH domain, such as *UBP16* in *Arabidopsis* (Zhou et al., 2012), and *ZmUBP15*, *ZmUBP16* and *ZmUBP19* in maize (Kong et al., 2019), have been reported to encoding ubiquitin-specific protease for salt-tolerance. In addition, in this study we observed five ubiquitin families were upregulated. The up-regulation of these ubiquitin-specific protease genes is consistent with the enhancement of ubiquitination in protein involved in salt tolerance in Adina. Furthermore, all of the 13 glycosyl transferases (*GTs*), enzymes in charge of glycosylation, were upregulated after salt stress treatment. GTs, as well the UDP-sugar glycosyltransferases (UGTs), have been illustrated to have crucial roles in improving plant resistance to stress conditions (Oussama et al., 2015; Pasquet et al., 2016). The enhanced expression of the *GT*s in our study suggests an advanced glycosylation process in alfalfa, in both Adina and Zhaodong, in order for the plants to achieve stronger salt stress adaptation, which is consistent with the reported salt-tolerance roles of *UGT85A5* (Sun et al., 2013), *UGT76E11* (Qin et al., 2018), and *CrUGT87A1* (Zhang et al., 2020b). We also identified 21 methyltransferases (*MT*), the enzyme that catalyze the methylation reaction. Recent studies suggested that the two Caffeic acid O-methyltransferase (*COMT*) genes, *CrCOMT* (Zhang et al., 2019) and *SlCOMT1* (Sun et al., 2020), had the higher transformation of N-acetylserotonin into melatonin, leading to higher salt tolerance. The regulated methylation processes under salt stress conditions might be an important salt tolerance mechanism in alfalfa. Seven sulfotransferases (*SOT*), enzymes catalyzing the sulfation process, were also increased after salt stress both in Adina and Zhaodong. The changes of the *SOT*s in our study are consistent with what is documented in transcriptional assays, such as the expression of *AtSOT12* (Hirschmann et al., 2014), *BrSOT*s (Jin et al., 2019) and *SjSOT*s (Lu et al., 2020) after exposure to salt stress. A leading hypothesis is that the sulfation of polysaccharides that causes the modification of the composition of cell walls is the main reason for increased salt tolerance in plants (Hirschmann et al., 2014). The specific salt adaptation roles of *SOT*s in alfalfa need to be further explored.

## Conclusions

In this study, we conducted a time-course transcriptome assay at four and eight hours after exposure to high salinity to explore the integrative molecular salt stress adaptation mechanisms in alfalfa. DEGs identified from the RNA-seq were analyzed with GO annotation and KEGG pathway enrichment database, in order to highlight the functional roles of transcription factor activity, signal sensing and transduction, and catalytic function. DEGs involved in or encoding ion and membrane homeostasis, Ca^2+^ sensing and transduction, phytohormone signaling and regulation, transcription factors, antioxidation process, and post-translational modification, are shown to contributing to salt stress adaptation in alfalfa. Our transcriptome results widen the knowledge of the molecular regulatory mechanisms in alfalfa and provide a number of candidate functional genes for salt tolerance breeding and improvements in alfalfa.

## Data availability statement

The original contributions presented in the study are publicly available. This data can be found here: NCBI, PRJNA821982.

## Author contributions

DM, JC, and QM were responsible for the experimental design. DM, WW, and LZ performed the experiments. DM, JL, and LS prepared the manuscript and coordinated its revision. DM and JC read and revised the manuscript. All authors contributed to the article and approved the submitted version.
