# Supplementary material for: Comparative time-course transcriptome analysis of two contrasting alfalfa (Medicago sativa L.) genotypes reveals tolerance mechanisms to salt stress
Source: Front Plant Sci. 2022 Dec 8;13:1070846. doi: 10.3389/fpls.2022.1070846 (PMC9773191; doi:10.3389/fpls.2022.1070846)
Supplement: Supplementary file 1 [file DataSheet_1.docx]

**Table S1 Primers used in quantitative RT-PCR validation of differentially expressed genes**

| Gene ID | Primers (5'-3') |
| --- | --- |
| Cluster-171808.262653 | F: GCCACAGCGACATCCACCAAG |
|  | R: CGGAGCCGACTTCAGTGACAATG |
| Cluster-171808.201554 | F: AGCCATATGCGCTCTCATTCCTC |
|  | R: CATCGAGCAGGCTGGAAGCTATAC |
| Cluster-171808.190107 | F: ACTGAACTCATACTCCAACAAAAATCA |
|  | R: GATTGCAATGTATGTTGTTGTCTTAGTC |
| Cluster-171808.189526 | F: GCTACTCTCGACTGGAAACGG |
|  | R: GGTGGAGTCAGGGTAGTGGA |
| Cluster-171808.198991 | F: TGATGGTAGAAGGCTGCAATGGC |
|  | R: AGTCACTGGCACTGGCATCAATC |
| Cluster-171808.116680 | F: AATCTGAGGAGTGCCACGAGGAG |
|  | R: TTCACGACCTACGCCGACTACC |
| Cluster-171808.81350 | F: CCTCGGAAGTGGCAGAATACGC |
|  | R: GTCTCTGGTTCAGGTTCGACTGTG |
| β-Actin | F: CAAAAGATGGCAGATGCTGAGGAT |
|  | R: CATGACACCAGTATGACGAGGTCG |

**Table S2 Quality assessment of sequencing**

| Sample | Raw Reads | Clean reads | Clean bases | Error(%) | Q20(%) | Q30(%) | GC(%) |
| --- | --- | --- | --- | --- | --- | --- | --- |
| N0-1 | 46477714 | 44496844 | 6.67G | 0.02 | 95.8 | 89.72 | 50.07 |
| N0-2 | 47052088 | 46087948 | 6.91G | 0.02 | 96.85 | 92.23 | 48.84 |
| N0-3 | 50500998 | 48353338 | 7.25G | 0.02 | 95.85 | 89.86 | 42.36 |
| N4-1 | 50561496 | 48045052 | 7.21G | 0.02 | 95.76 | 89.57 | 53.37 |
| N4-2 | 58608376 | 56045638 | 8.41G | 0.02 | 96.08 | 90.29 | 44.49 |
| N4-3 | 51211614 | 49974002 | 7.5G | 0.02 | 97.14 | 92.91 | 41.74 |
| N8-1 | 58533032 | 56760774 | 8.51G | 0.02 | 97.02 | 92.55 | 55.56 |
| N8-2 | 45493654 | 43613062 | 6.54G | 0.02 | 96.04 | 90.26 | 42.96 |
| N8-3 | 47912900 | 45918852 | 6.89G | 0.02 | 96.62 | 91.45 | 42 |
| M0-1 | 54166240 | 51916048 | 7.79G | 0.02 | 96.26 | 90.74 | 46.04 |
| M0h-2 | 56575026 | 54517856 | 8.18G | 0.02 | 96.1 | 90.47 | 43.6 |
| M0h-3 | 47852746 | 46467558 | 6.97G | 0.02 | 96.28 | 91.3 | 43.1 |
| M4h-1 | 50750774 | 47585828 | 7.14G | 0.02 | 96.28 | 90.72 | 41.4 |
| M4h-2 | 54837854 | 51469248 | 7.72G | 0.02 | 96.01 | 90.07 | 41.99 |
| M4h-3 | 53007748 | 51712258 | 7.76G | 0.02 | 96.9 | 92.51 | 42.22 |
| M8h-1 | 48357056 | 47230310 | 7.08G | 0.02 | 96.9 | 92.37 | 42.05 |
| M8h-2 | 52583740 | 50449760 | 7.57G | 0.02 | 96.22 | 90.67 | 45.09 |
| M8h-3 | 51540284 | 49002276 | 7.35G | 0.02 | 95.71 | 89.71 | 44.05 |

**Table S3 The expression changes and PFAM descriptions of the Candidates DEGs**

| **Gene ID** | **N4** | **N8** | **M4** | **M8** | **PFAM ID** | **PFAM description** |  |  |
| --- | --- | --- | --- | --- | --- | --- | --- | --- |
| **Ion and membrane homeostasis** | | | |  |  |  |  |  |
| Cluster-171808.59136 | 21.854 | - | - | - | PF00005 | ABC transporter |  |  |
| Cluster-118040.0 | 21.884 | - | - | - | PF00005 | ABC transporter |  |  |
| Cluster-171808.22602 | 21.884 | - | - | - | PF00005 | ABC transporter |  |  |
| Cluster-87623.1 | 21.884 | - | - | - | PF00005 | ABC transporter |  |  |
| Cluster-171808.22602 | 21.884 | - | - | - | PF00005 | ABC transporter |  |  |
| Cluster-85318.0 | -21.008 | - | - | - | PF00005 | ABC transporter |  |  |
| Cluster-629.0 | - | 22.817 | - | - | PF00005 | ABC transporter |  |  |
| Cluster-171808.22602 | - | 22.412 | - | - | PF00005 | ABC transporter |  |  |
| Cluster-171808.67714 | - | 22.909 | - | - | PF00005 | ABC transporter |  |  |
| Cluster-178530.0 | - | 22.918 | - | - | PF00005 | ABC transporter |  |  |
| Cluster-21212.1 | - | 22.97 | - | - | PF00664 | ABC transporter |  |  |
| Cluster-171808.137833 | - | - | 4.2861 | - | PF00005 | ABC transporter |  |  |
| Cluster-171808.255655 | - | 23.121 | - | - | PF01061 | ABC-2 type transporter |  |  |
| Cluster-171808.232906 | - | - | 5.2276 | - | PF01061 | ABC-2 type transporter |  |  |
| Cluster-171808.249986 | - | - | 7.6001 | - | PF01061 | ABC-2 type transporter |  |  |
| Cluster-171808.12255 | - | 22.707 | - | - | PF16326 | ABC transporter C-terminal domain |  |  |
| Cluster-171808.73937 | - | - | -4.7601 | - | PF16326 | ABC transporter C-terminal domain |  |  |
| Cluster-81296.2 | 22.139 | 22.363 | - | - | PF00654 | Voltage gated chloride channel |  |  |
| Cluster-4057.0 | 22.242 | - | - | - | PF00654 | Voltage gated chloride channel |  |  |
| Cluster-171808.106123 | - | - | -4.6978 | - | PF00654 | Voltage gated chloride channel |  |  |
| Cluster-156046.0 | 22.32 | 23.13 | - | - | PF01699 | Sodium/calcium exchanger protein |  |  |
| Cluster-192125.1 | 21.957 | - | - | - | PF01699 | Sodium/calcium exchanger protein |  |  |
| Cluster-938.1 | - | 22.338 | - | - | PF01699 | Sodium/calcium exchanger protein |  |  |
| Cluster-171808.71564 | - | 22.584 | - | - | PF03553 | Na+/H+ antiporter family |  |  |
| **Ca^2+^ sensing and transduction** | | | |  |  |  |  |  |
| Cluster-171808.29077 | 22.182 | - | - | - | PF03185 | Calcium-activated potassium channel, beta subunit |  |  |
| Cluster-171808.117678 | 22.331 | - | - | - | PF03185 | Calcium-activated potassium channel, beta subunit |  |  |
| Cluster-171808.288624 | - | - | 8.4255 | - | PF03493 | Calcium-activated BK potassium channel alpha subunit |  |  |
| Cluster-171808.73702 | - | 23.202 | - | - | PF13405 | EF-hand domain |  |  |
| Cluster-171808.167305 | - | 22.904 | - | - | PF13405 | EF-hand domain |  |  |
| Cluster-171808.7730 | - | 22.707 | - | - | PF13499 | EF-hand domain pair |  |  |
| Cluster-171808.267258 | - | -21.427 | - | - | PF13202 | EF hand |  |  |
| Cluster-21934.1 | - | -21.138 | - | - | PF13202 | EF hand |  |  |
| Cluster-171808.26029 | - | 22.234 | - | - | PF13202 | EF hand |  |  |
| Cluster-1732.0 | - | 22.412 | - | - | PF00036 | EF hand |  |  |
| Cluster-171808.196249 | - | - | -4.6936 | - | PF13405 | EF-hand domain |  |  |
| Cluster-171808.131326 | - | - | -7.692 | - | PF13499 | EF-hand domain pair |  |  |
| Cluster-171808.272285 | - | - | -4.8656 | - | PF13499 | EF-hand domain pair |  |  |
| Cluster-171808.211004 | - | - | -5.2472 | - | PF13202 | EF hand |  |  |
| Cluster-171808.147372 | - | - | -7.6286 | - | PF13202 | EF hand |  |  |
| Cluster-171808.122921 | - | - | -7.4069 | - | PF07887 | Calmodulin binding protein-like |  |  |
| **Phytohormone signaling and regulation** | | | | |  |  |  |  |
| Cluster-5535.2 | - | 22.178 | - | - | PF13371 | Tetratricopeptide repeat |  |  |
| Cluster-171808.33813 | - | 22.707 | - | - | PF13371 | Tetratricopeptide repeat |  |  |
| Cluster-171808.23685 | - | 22.547 | - | - | PF13181 | Tetratricopeptide repeat |  |  |
| Cluster-8309.0 | - | 22.234 | - | - | PF13176 | Tetratricopeptide repeat |  |  |
| Cluster-171808.280282 | - | -21.071 | - | - | PF13371 | Tetratricopeptide repeat |  |  |
| Cluster-171808.123774 | - | 22.547 | - | - | PF13371 | Tetratricopeptide repeat |  |  |
| Cluster-171808.33751 | - | 22.563 | - | - | PF13374 | Tetratricopeptide repeat |  |  |
| Cluster-171808.311428 | - | 22.387 | - | - | PF13374 | Tetratricopeptide repeat |  |  |
| Cluster-209805.0 | - | 22.563 | - | - | PF13371 | Tetratricopeptide repeat |  |  |
| Cluster-171808.310760 | - | 23.13 | - | - | PF00515 | Tetratricopeptide repeat |  |  |
| Cluster-171808.15667 | - | 22.338 | - | - | PF07721 | Tetratricopeptide repeat |  |  |
| Cluster-8151.0 | - | 22.526 | - | - | PF13371 | Tetratricopeptide repeat |  |  |
| Cluster-171808.71214 | - | 22.707 | - | - | PF13414 | TPR repeat |  |  |
| Cluster-171808.121932 | - | 22.234 | - | - | PF13414 | TPR repeat |  |  |
| Cluster-171808.293446 | - | - | 7.2902 | - | PF07721 | Tetratricopeptide repeat |  |  |
| Cluster-171808.152501 | - | - | -4.5288 | - | PF13374 | Tetratricopeptide repeat |  |  |
| Cluster-171808.79493 | - | - | -8.4937 | - | PF13371 | Tetratricopeptide repeat |  |  |
| Cluster-171808.191903 | - | - | 5.6517 | - | PF00515 | Tetratricopeptide repeat |  |  |
| Cluster-171808.191044 | - | - | -8.3974 | - | PF00515 | Tetratricopeptide repeat |  |  |
| Cluster-171808.212806 | - | - | -9.0155 | - | PF00515 | Tetratricopeptide repeat |  |  |
| Cluster-171808.115809 | - | - | -9.059 | - | PF13174 | Tetratricopeptide repeat |  |  |
| Cluster-85862.1 | 22.182 | - | - | - | PF12937 | F-box-like |  |  |
| Cluster-168137.2 | 21.854 | - | - | - | PF12937 | F-box-like |  |  |
| Cluster-171808.98194 | 22.331 | - | - | - | PF12937 | F-box-like |  |  |
| Cluster-171808.42110 | -21.191 | - | - | - | PF00646 | F-box domain |  |  |
| Cluster-171808.310065 | - | 22.435 | - | - | PF00646 | F-box domain |  |  |
| Cluster-171808.42110 | - | -21.261 | - | - | PF00646 | F-box domain |  |  |
| Cluster-171808.267692 | - | 22.952 | - | - | PF15966 | F-box |  |  |
| Cluster-171808.198468 | - | - | -7.3465 | - | PF12937 | F-box-like |  |  |
| Cluster-171808.185779 | - | - | -6.5554 | - | PF12937 | F-box-like |  |  |
| Cluster-171808.36101 | - | - | -7.7174 | - | PF00646 | F-box domain |  |  |
| Cluster-171808.124518 | - | - | -6.9551 | - | PF15966 | F-box |  |  |
| Cluster-171808.21154 | - | 22.918 | - | - | PF13855 | Leucine rich repeat |  |  |
| Cluster-171808.21157 | - | 22.313 | - | - | PF13855 | Leucine rich repeat |  |  |
| Cluster-171808.21156 | - | 22.178 | - | - | PF00560 | Leucine rich repeat |  |  |
| Cluster-80517.0 | - | 22.584 | - | - | PF13855 | Leucine rich repeat |  |  |
| Cluster-171808.58031 | - | 22.631 | - | - | PF00560 | Leucine rich repeat |  |  |
| Cluster-172939.0 | - | 22.458 | - | - | PF13855 | Leucine rich repeat |  |  |
| Cluster-171808.292908 | - | 22.234 | - | - | PF13516 | Leucine rich repeat |  |  |
| Cluster-171808.183490 | - | 22.666 | - | - | PF13855 | Leucine rich repeat |  |  |
| Cluster-1791.0 | - | 22.481 | - | - | PF00560 | Leucine rich repeat |  |  |
| Cluster-261165.1 | - | 22.435 | - | - | PF00560 | Leucine rich repeat |  |  |
| Cluster-89743.2 | - | 22.666 | - | - | PF13855 | Leucine rich repeat |  |  |
| Cluster-131295.0 | - | 23.121 | - | - | PF13855 | Leucine rich repeat |  |  |
| Cluster-171808.212654 | - | - | -8.3104 | - | PF00560 | Leucine rich repeat |  |  |
| Cluster-171808.202006 | - | - | -8.948 | - | PF13516 | Leucine rich repeat |  |  |
| Cluster-171808.168422 | - | - | -5.3168 | - | PF13516 | Leucine rich repeat |  |  |
| Cluster-171808.102062 | - | - | 7.5752 | - | PF00560 | Leucine rich repeat |  |  |
| Cluster-171808.219157 | - | - | -4.3782 | - | PF13855 | Leucine rich repeat |  |  |
| Cluster-171808.159169 | - | - | -8.4025 | - | PF13855 | Leucine rich repeat |  |  |
| Cluster-171808.40089 | - | - | -7.8058 | - | PF13855 | Leucine rich repeat |  |  |
| Cluster-171808.120214 | - | - | -7.8119 | - | PF13855 | Leucine rich repeat |  |  |
| Cluster-171808.249581 | - | - | 7.4611 | - | PF00481 | Protein phosphatase 2C |  |  |
| Cluster-171808.99889 | - | - | 6.771 | - | PF00481 | Protein phosphatase 2C |  |  |
| Cluster-171808.166658 | - | - | 4.7287 | - | PF00481 | Protein phosphatase 2C |  |  |
| Cluster-171808.228475 | - | - | 7.0756 | - | PF00481 | Protein phosphatase 2C |  |  |
| Cluster-171808.72438 | - | - | -7.7537 | - | PF00481 | Protein phosphatase 2C |  |  |
| Cluster-171808.180490 | - | - | 6.7543 | - | PF00481 | Protein phosphatase 2C |  |  |
| Cluster-171808.98852 | - | - | 7.7685 | - | PF00481 | Protein phosphatase 2C |  |  |
| Cluster-171808.226574 | - | - | 4.5689 | - | PF00481 | Protein phosphatase 2C |  |  |
| **Transcription factors** | | |  |  |  |  |  |  |
| Cluster-171808.306942 | - | 22.287 | - | - | PF16622 | Zinc finger, C2H2 type |  |  |
| Cluster-150859.0 | - | 22.435 | - | - | PF16622 | Zinc finger, C2H2 type |  |  |
| Cluster-171808.307396 | - | 22.745 | - | - | PF00096 | Zinc finger, C2H2 type |  |  |
| Cluster-171808.129255 | 21.979 | - | - | - | PF00096 | Zinc finger, C2H2 type |  |  |
| Cluster-171808.281662 | 21.854 | - | - | - | PF13912 | Zinc finger, C2H2 type |  |  |
| Cluster-136494.0 | 22.05 | - | - | - | PF16588 | Zinc finger, C2H2 type |  |  |
| Cluster-171808.240696 | - | - | -7.9039 | - | PF16588 | Zinc finger, C2H2 type |  |  |
| Cluster-171808.39167 | -19.888 | - | - | - | PF00096 | Zinc finger, C2H2 type |  |  |
| Cluster-85279.0 | 21.957 | - | - | - | PF16622 | Zinc finger, C2H2 type |  |  |
| Cluster-171808.130033 | - | 22.666 | - | - | PF00097 | Zinc finger, C3HC4 type (RING finger) |  |  |
| Cluster-171808.293526 | 22.353 | - | - | - | PF00097 | Zinc finger, C3HC4 type (RING finger) |  |  |
| Cluster-171808.292509 | 21.775 | 22.313 | - | - | PF14634 | Zinc-RING finger domain |  |  |
| Cluster-188467.1 | 22.05 | 22.763 | - | - | PF11722 | CCCH zinc finger |  |  |
| Cluster-171808.186309 | - | 22.707 | - | - | PF00642 | Zinc finger C-x8-C-x5-C-x3-H type (and similar) |  |  |
| Cluster-171808.155512 | - | - | 8.3398 | 10.412 | PF00098 | Zinc finger CCHC type Zinc knuckle |  |  |
| Cluster-171808.299966 | 21.825 | 22.631 | - | - | PF00098 | Zinc finger CCHC type Zinc knuckle |  |  |
| Cluster-1014.0 | - | 22.631 | - | - | PF00098 | Zinc finger CCHC type Zinc knuckle |  |  |
| Cluster-171808.299966 | - | 22.631 | - | - | PF00098 | Zinc finger CCHC type Zinc knuckle |  |  |
| Cluster-171808.299966 | 21.825 |  | - | - | PF00098 | Zinc finger CCHC type Zinc knuckle |  |  |
| Cluster-171808.62262 | 22.05 |  | - | - | PF00643 | B-box zinc finger |  |  |
| Cluster-122061.1 | - | 22.287 | - | - | PF00643 | B-box zinc finger |  |  |
| Cluster-171808.79342 | - | 22.412 | - | - | PF00643 | B-box zinc finger |  |  |
| Cluster-107174.0 | - | 22.97 | - | - | PF00320 | GATA zinc finger |  |  |
| Cluster-171808.102355 | - | 22.206 | - | - | PF00320 | GATA zinc finger |  |  |
| Cluster-171808.75880 | - | - | -8.1638 | - | PF04690 | YABBY protein |  |  |
| Cluster-171808.193067 | - | - | -7.2561 | - | PF04690 | YABBY protein |  |  |
| Cluster-171808.164296 | - | 22.261 | - | - | PF03110 | SBP domain |  |  |
| Cluster-171808.216121 | - | 22.952 | - | - | PF03110 | SBP domain |  |  |
| **Antioxidation process** | | |  |  |  |  |  |  |
| Cluster-171808.22832 | 22.663 | - | - | - | PF13409 | Glutathione S-transferase, N-terminal domain |  |  |
| Cluster-241633.0 | - | - | -8.2593 | - | PF13417 | Glutathione S-transferase, N-terminal domain |  |  |
| Cluster-171808.119104 | - | - | 7.7391 | - | PF13409 | Glutathione S-transferase, N-terminal domain |  |  |
| Cluster-171808.243442 | - | - | 7.3009 | - | PF02798 | Glutathione S-transferase, N-terminal domain |  |  |
| Cluster-171808.79798 | - | 22.987 | - | - | PF07992 | Pyridine nucleotide-disulphide oxidoreductase |  |  |
| Cluster-132696.0 | - | 22.504 | - | - | PF00070 | Pyridine nucleotide-disulphide oxidoreductase |  |  |
| Cluster-171808.7073 | - | 22.952 | - | - | PF07992 | Pyridine nucleotide-disulphide oxidoreductase |  |  |
| Cluster-171808.6854 | - | 22.946 | - | - | PF00070 | Pyridine nucleotide-disulphide oxidoreductase |  |  |
| Cluster-246597.1 | - | 22.287 | - | - | PF07992 | Pyridine nucleotide-disulphide oxidoreductase |  |  |
| Cluster-111.0 | - | 22.853 | - | - | PF07992 | Pyridine nucleotide-disulphide oxidoreductase |  |  |
| Cluster-171808.115943 | - | 22.547 | - | - | PF07992 | Pyridine nucleotide-disulphide oxidoreductase |  |  |
| Cluster-136461.0 | - | 22.178 | - | - | PF07992 | Pyridine nucleotide-disulphide oxidoreductase |  |  |
| Cluster-171808.181582 | - | - | -10.905 | - | PF07992 | Pyridine nucleotide-disulphide oxidoreductase |  |  |
| Cluster-171808.169231 | - | - | -5.3566 | - | PF07992 | Pyridine nucleotide-disulphide oxidoreductase |  |  |
| Cluster-171808.198319 | - | - | -4.3174 | - | PF02852 | Pyridine nucleotide-disulphide oxidoreductase |  |  |
| Cluster-171808.82571 | - | 22.666 |  | - | PF00171 | Aldehyde dehydrogenase family |  |  |
| Cluster-171808.28676 | - | 22.363 |  | - | PF00171 | Aldehyde dehydrogenase family |  |  |
| Cluster-90478.0 | - | 22.287 |  | - | PF00171 | Aldehyde dehydrogenase family |  |  |
| Cluster-171808.150849 | - | - | -7.3476 | - | PF00171 | Aldehyde dehydrogenase family |  |  |
| Cluster-171808.220150 | - | - | 5.2788 | - | PF00171 | Aldehyde dehydrogenase family |  |  |
| Cluster-171808.199795 | - | - | -4.6601 | - | PF00171 | Aldehyde dehydrogenase family |  |  |
| Cluster-171808.232038 | - | - | 5.0611 | - | PF00171 | Aldehyde dehydrogenase family |  |  |
| **Post-translational modification** | | | |  |  |  |  |  |
| Cluster-171808.221888 | 22.634 | 22.952 | - | - | PF00443 | Ubiquitin carboxyl-terminal hydrolase |  |  |
| Cluster-171808.18583 | 22.32 | 22.313 | - | - | PF00443 | Ubiquitin carboxyl-terminal hydrolase |  |  |
| Cluster-122272.0 | - | 22.178 | - | - | PF00443 | Ubiquitin carboxyl-terminal hydrolase |  |  |
| Cluster-122235.1 | - | 21.213 | - | - | PF00443 | Ubiquitin carboxyl-terminal hydrolase |  |  |
| Cluster-171808.236746 | - | - | -8.844 | - | PF00443 | Ubiquitin carboxyl-terminal hydrolase |  |  |
| Cluster-124433.0 | 22.004 | 23.121 | - | - | PF00240 | Ubiquitin family |  |  |
| Cluster-179314.0 | - | 22.726 | - | - | PF00240 | Ubiquitin family |  |  |
| Cluster-171808.311101 | - | 22.745 | - | - | PF00240 | Ubiquitin family |  |  |
| Cluster-182266.1 | - | 22.338 | - | - | PF00240 | Ubiquitin family |  |  |
| Cluster-171808.100763 | - | - | 8.1389 | - | PF00240 | Ubiquitin family |  |  |
| Cluster-184938.0 | 22.301 | - | - | - | PF01501 | Glycosyl transferase family 8 |  |  |
| Cluster-171808.34255 | 22.094 | 22.835 | - | - | PF13506 | Glycosyl transferase family 21 |  |  |
| Cluster-171808.13353 | - | 22.234 | - | - | PF04101 | Glycosyltransferase family 28 |  |  |
| Cluster-145698.0 | - | 22.206 | - | - | PF00982 | Glycosyltransferase family 20 |  |  |
| Cluster-171808.230363 | - | - | 4.0658 | - | PF04101 | Glycosyltransferase family 28 |  |  |
| Cluster-171808.193654 | - | - | 5.8225 | - | PF01501 | Glycosyl transferase family 8 |  |  |
| Cluster-171808.250399 | - | - | 7.7276 | - | PF01501 | Glycosyl transferase family 8 |  |  |
| Cluster-1606.0 | - | 22.313 | - | - | PF00201 | UDP-glucoronosyl and UDP-glucosyl transferase |  |  |
| Cluster-171808.299342 | - | 22.987 | - | - | PF00201 | UDP-glucoronosyl and UDP-glucosyl transferase |  |  |
| Cluster-1242.0 | - | 22.526 | - | - | PF00201 | UDP-glucoronosyl and UDP-glucosyl transferase |  |  |
| Cluster-171808.299342 | 22.094 | 22.987 | - | - | PF00201 | UDP-glucoronosyl and UDP-glucosyl transferase |  |  |
| Cluster-171808.249499 | - | - | 9.425 | - | PF00201 | UDP-glucoronosyl and UDP-glucosyl transferase |  |  |
| Cluster-171808.104616 | - | - | 6.9077 | - | PF00201 | UDP-glucoronosyl and UDP-glucosyl transferase |  |  |
| Cluster-96856.0 | -21.135 | - | - | - | PF05175 | Methyltransferase small domain |  |  |
| Cluster-226192.0 | 22.028 | - | - | - | PF05175 | Methyltransferase small domain |  |  |
| Cluster-171808.241430 | -11.407 | - | - | - | PF08241 | Methyltransferase domain |  |  |
| Cluster-171808.284393 | -21.23 | - | - | - | PF08241 | Methyltransferase domain |  |  |
| Cluster-171808.15010 | - | 22.178 | - | - | PF05175 | Methyltransferase small domain |  |  |
| Cluster-171808.4821 | - | 22.481 | - | - | PF05175 | Methyltransferase small domain |  |  |
| Cluster-171808.23298 | - | 22.338 | - | - | PF05175 | Methyltransferase small domain |  |  |
| Cluster-127988.0 | - | 22.481 | - | - | PF08241 | Methyltransferase domain |  |  |
| Cluster-171808.284393 | - | -21.318 | - | - | PF08241 | Methyltransferase domain |  |  |
| Cluster-150785.1 | - | 22.97 | - | - | PF08241 | Methyltransferase domain |  |  |
| Cluster-125687.3 | - | 22.981 | - | - | PF02390 | Putative methyltransferase |  |  |
| Cluster-171808.181651 | - | - | -5.3026 | - | PF05175 | Methyltransferase small domain |  |  |
| Cluster-171808.167748 | - | - | 9.5228 | - | PF08241 | Methyltransferase domain |  |  |
| Cluster-171808.70639 | - | 22.458 | - | - | PF00891 | O-methyltransferase |  |  |
| Cluster-171808.103898 | - | - | 12.465 | - | PF00891 | O-methyltransferase |  |  |
| Cluster-171808.122379 | - | - | -7.6443 | - | PF00891 | O-methyltransferase |  |  |
| Cluster-171808.179975 | - | - | 7.4289 | - | PF00891 | O-methyltransferase |  |  |
| Cluster-171808.103007 | - | - | 13.333 | - | PF00891 | O-methyltransferase |  |  |
| Cluster-171808.160209 | - | - | 12.22 | - | PF00891 | O-methyltransferase |  |  |
| Cluster-171808.172838 | - | - | 10.323 | - | PF00891 | O-methyltransferase |  |  |
| Cluster-171808.103816 | - | - | 10.15 | - | PF00891 | O-methyltransferase |  |  |
| Cluster-181143.3 | - | 22.563 | - | - | PF03567 | Sulfotransferase family |  |  |
| Cluster-171808.7885 | - | 22.969 | - | - | PF00685 | Sulfotransferase domain |  |  |
| Cluster-171808.271607 | - | 22.178 | - | - | PF00685 | Sulfotransferase domain |  |  |
| Cluster-171808.100510 | - | - | 6.8129 | - | PF03567 | Sulfotransferase family |  |  |
| Cluster-171808.250822 | - | - | 6.4549 | - | PF00685 | Sulfotransferase domain |  |  |
| Cluster-171808.101176 | - | - | 8.3721 | - | PF00685 | Sulfotransferase domain |  |  |
| Cluster-171808.250822 | - | - | 6.4549 | - | PF00685 | Sulfotransferase domain |  |  |

N4, N8, represent the comparison groups of the genes identified in 4 h and 8 h’s treatments to 0 h in Adina, respectively. M4, M8, represent the comparison groups of the genes identified in 4 h and 8 h’s treatments to 0 h in Zhaodong, respectively. The log2 (fold change) value of each gene is demonstrated in N4, N8, M4, and M8 columns to represent the expression change after salt stress.
