# Supplementary material for: Comparative time-course transcriptome analysis of two contrasting alfalfa (Medicago sativa L.) genotypes reveals tolerance mechanisms to salt stress
Source: Front Plant Sci. 2022 Dec 8;13:1070846. doi: 10.3389/fpls.2022.1070846 (PMC9773191; doi:10.3389/fpls.2022.1070846)
Supplement: Supplementary file 2 [file DataSheet_2.docx]

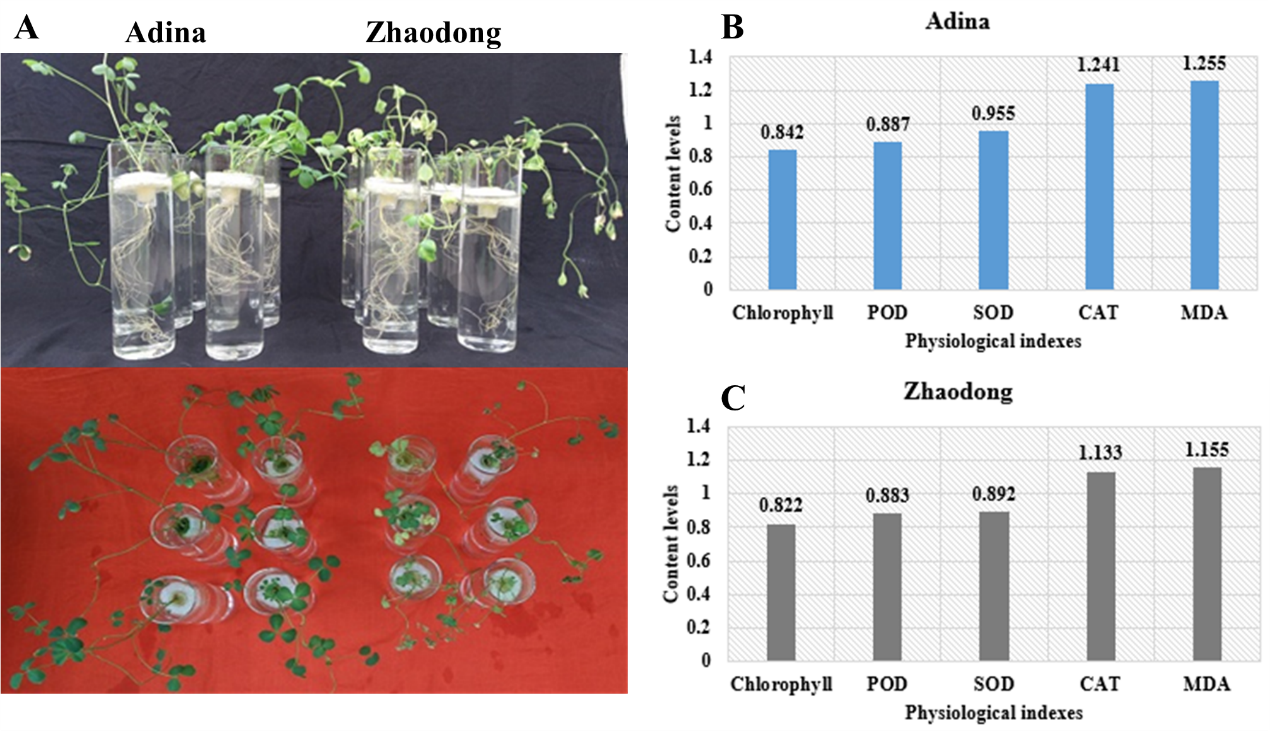


**Figure S1.** Growth and physiological performance of Adina and Zhaodong under salt stress. Growth phenotype of Adina and Zhaodong under salt stress (A), physiological indexes of Adina (B) and Zhaodong (C) after salt stress. Alfalfa Adina plants were treated with 300 mM NaCl for 5 days, and Zhaodong 150 mM 5 days.


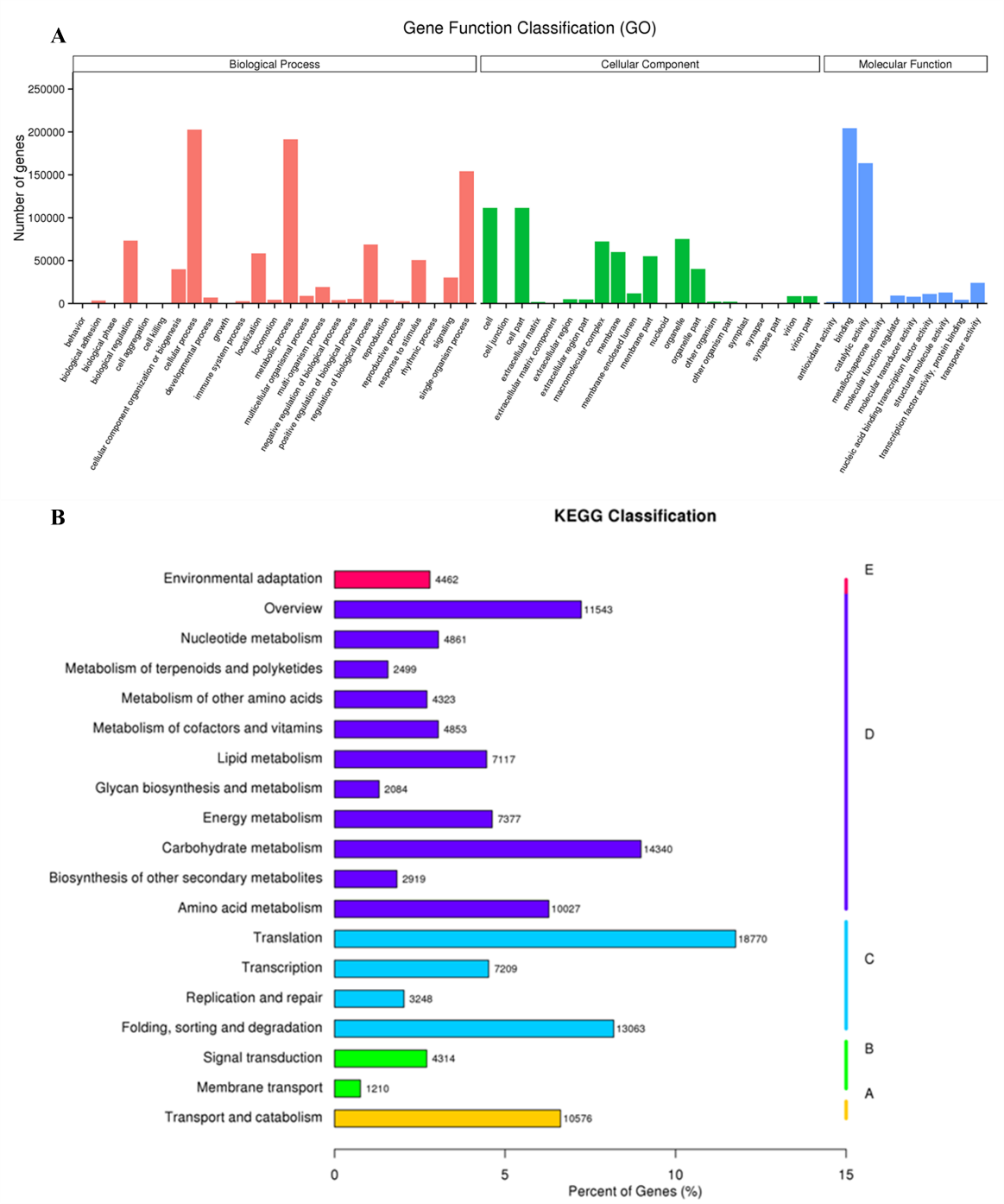


**Figure. S2** Unigenes GO annotations and KEGG pathway assignments. (A) Gene ontology (GO) categorization of assembled unigenes, (B) Kyoto encyclopedia of genes and genomes (KEGG) classification of assembled unigenes.
